# In-utero HIV exposure and cardiometabolic health among children 5–8 years: findings from a prospective birth cohort in South Africa

**DOI:** 10.1097/QAD.0000000000003412

**Published:** 2022-10-19

**Authors:** Angela M. Bengtson, Jennifer Pellowski, Stephen McGarvey, Rae McGinty, Maresa Botha, Tiffany Burd, David Burgner, Toby Mansell, Heather J. Zar

**Affiliations:** a Department of Epidemiology; b International Health Institute; c Department of Behavior and Social Sciences, Brown University School of Public Health, Providence, Rhode Island, USA; d Division of Epidemiology and Biostatistics, School of Public Health and Family Medicine, University of Cape Town; e Department of Paediatrics and Child Health, Red Cross War Memorial Children's Hospital; f South Africa Medical Research Council Unit on Child and Adolescent Health, University of Cape Town, Cape Town, South Africa; g Murdoch Children's Research Institute, Royal Children's Hospital; h Department of Paediatrics, University of Melbourne, Parkville, Victoria, Australia.

**Keywords:** blood pressure, body composition, cardiometabolic health, glucose metabolism, HIV exposure, lipids

## Abstract

To evaluate if in-utero HIV exposure is associated with adverse cardiometabolic health outcomes at 5–8 years of age.

**Design:**

Prospective cohort study.

**Methods:**

We enrolled a random sample of HIV-exposed but uninfected (HEU) and HIV-unexposed children from the Drakenstein Child Health study, a longitudinal birth cohort study in Cape Town, South Africa, in a cardiometabolic health pilot study. Outcomes were assessed by trained study staff and included: anthropometry, body composition and size, blood pressure, fasting plasma glucose, HbA1c, lipids, and insulin resistance using HOMA-IR. We used multivariable linear and log-binomial regression to estimate associations between HIV-exposure and cardiometabolic outcomes, adjusted for child age, sex, height, body size, and maternal factors as appropriate.

**Results:**

We included 260 children (HEU *n* = 100, HIV-unexposed *n* = 160). HEU children had older mothers (median age 30 vs. 26 years), with minimal differences in gestational age and size at birth by HIV-exposure status. In multivariable analyses, HEU children had lower weight-for-age (mean difference −0.35, 95% confidence interval −0.66, −0.05), and height-for-age (mean difference −0.29, 95% confidence interval −0.56, −0.03; *z*-scores). There were no differences in adiposity, impaired glucose metabolism, or lipid levels by HIV-exposure status. Overall, 12% of children had blood pressure more than 90th percentile, with no differences by HIV-exposure status.

**Conclusion:**

Overall, there were few differences in cardiometabolic outcomes between HEU and HIV-unexposed children in this South African cohort. Although these findings are reassuring, monitoring of cardiometabolic health is important as HEU and HIV-unexposed children enter adolescence and cardiometabolic risk trajectories become established.

## Introduction

Since 2010, the number of infants who are perinatally HIV-infected has fallen by more than half with the expansion of antiretroviral therapy (ART) for pregnant women living with HIV [1]. In parallel, the number of children exposed but uninfected with HIV (HEU) has increased to over 1.4 million born each year [2]. Nearly 90% of HEU children live in low and middle-income countries [3]. South Africa has the highest number of HEU children, with 3.5 million or 24% of all HEU children worldwide [2]. Despite not living with HIV, HEU children have altered immune function and experience higher levels of morbidity in early childhood compared with HIV-unexposed children, which may influence their long-term health [2,4–10].

In particular, HEU children may be at increased risk for adverse cardiometabolic outcomes. HEU children have long been noted to have suboptimal growth compared with HIV-unexposed children [11–14]. Despite the lower weight, HIV exposure has been associated with higher levels of dyslipidemia in children [15,16]. HIV exposure has also been linked to an increased risk of obesity and hypertension in adolescents in the United States [17,18]. The mechanisms by which HIV-exposure influences cardiometabolic health are incompletely understood. However, emerging evidence demonstrates that HIV exposure and ART exposure *in utero* may alter metabolomic and lipidomic profiles [19–22], which could influence insulin resistance and glucose metabolism later in life [23]. Less is known about the clinical implications of HIV-associated and ART-associated metabolic changes, in part due to the differing maternal and social factors HEU and HIV-unexposed children experience that also influence cardiometabolic health [24,25]. To better understand the clinical impact of HIV exposure on children's cardiometabolic health, information from population-based cohorts of HEU and HIV-unexposed children from the same communities is urgently needed.

To address this gap, we investigated the associations between HIV-exposure status and several cardiometabolic outcomes at 5–8 years of age among children enrolled in a South African birth cohort.

## Methods

### Study population and design

We investigated cardiometabolic outcomes in children enrolled in the Drakenstein Child Health Study (DCHS), a longitudinal birth cohort study of HEU and HIV-unexposed children from the same periurban communities outside of Cape Town, South Africa [26]. The study community includes a stable population (e.g. little in-migration or out-migration) of approximately 200 000 and is characterized by high rates of poverty, substance use, and HIV infection [27]. Study communities have a well-established public healthcare system, which includes primary care, antenatal, obstetric, HIV, and prevention of mother-to-child transmission services free of charge in accordance with national guidelines [28].

Details of the DCHS have been published previously [26]. Briefly, pregnant women were recruited from two primary healthcare clinics, Mbekweni (serving a predominantly black African community) and TC Newman (serving a mixed ancestry community). Mothers were enrolled in the DCHS at 20–28 weeks’ gestation while attending routine antenatal care and were prospectively followed. Women were eligible for the study if they were 18 years or older, between 20 and 28 weeks’ gestation, planned attendance at one of the two recruitment clinics and intended to remain in the area [26,29]. Although racial and ethnic categories are sociocultural constructs, in our context use of these and related variables is useful in assessing and addressing ongoing health disparities. Between March 2012 and March 2015, 1225 pregnant women were enrolled into the DCHS; 88 (7.2%) mothers were lost to follow-up antenatally, and had a miscarriage or a stillbirth. In total, 1137 (*n* = 893 HIV-uninfected, *n* = 244 HIV-infected) women gave birth to 1143 live infants (four twins and one triplet). Nearly all (99%) of women living with HIV received ART during pregnancy in accordance with HIV guidelines at the time [30–33] and 70% had an undetectable viral load during pregnancy [29]. Women and their children were followed through delivery and at least annually thereafter [26].

As part of ongoing annual study visits, we conducted a pilot study to evaluate cardiometabolic outcomes among a random sample of 260 (*n* = 100 HEU and *n* = 160 HIV-unexposed) children in the DCHS cohort. Participants from the full DCHS cohort were eligible for inclusion into the pilot study if recruited and followed up at the Mbekweni study site (due to the higher prevalence of HEU children), and the child was not living with HIV (*n* = 2) [29,33]. Cardiometabolic assessments took place at an annual visit when children were 5–8 years of age and are hereafter referred to as the cardiometabolic visit. Ethical approval was obtained from the Faculty of Health Sciences Human Research Ethics Committee, University of Cape Town (401/2009), and the Western Cape Provincial Health Research committee. Mothers gave written informed consent at enrolment and assent was provided by children 7 years or older.

### Measures

The exposure of interest was HIV-exposure status during gestation for children. All women with unknown HIV status at study enrollment during pregnancy underwent HIV testing as part of routine care [28]. Women who tested negative for HIV at study enrollment were retested for HIV in the third trimester when feasible, and approximately every 3 months while breastfeeding as part of routine clinical care. HIV-exposed infants were given nevirapine syrup prophylaxis and tested for HIV at 6 weeks, 6–12 months, and 18 months of age, as part of routine care [28].

We investigated a range of cardiometabolic outcomes in children at 5–8 years of age. Body composition was evaluated by bioelectrical impedance analysis using the Tanita AB-140 device. The manufacturer's equations were used to estimate fat mass (in kilograms), percentage body fat, and percentage truncal fat at three study visits [34–36]. We selected the study visit with body composition data closest to the cardiometabolic visit, within 90 days (*n* = 252/260). Anthropometry, including height, weight, and triceps skinfold thickness, was assessed at each annual study visit by trained study staff using standardized protocols based on WHO guidelines and regularly calibrated equipment [37]. Continuous measures were converted to age-adjusted and sex-adjusted *z*-scores based on WHO child growth standards [38]. We also considered binary measures of low weight (BMIZ score <15th percentile) and high weight (BMIZ score >85th percentile) as secondary outcomes. Glucose metabolism and lipids were assessed using fasted blood samples. Diabetes was defined as fasting plasma glucose at least 7.0 mmol/l or HbA1c at least 6.5%, impaired fasting glucose as fasting plasma glucose at least 6.1 to less than 7.0 mmol/l, and prediabetes as HbA1c 5.7–6.4% [39,40]. Insulin resistance was estimated using the continuous Homeostatic Model Assessment (HOMA-IR) & binary indicator of HOMA-IR at least 3.0 [41,42]. Total cholesterol (TC), LDL cholesterol, HDL cholesterol, and triglycerides were evaluated as continuous measures and using established cut-points in children [43]. Blood pressure (BP) was measured after sitting in the clinic waiting room in a single arm using an electronic BP cuff by trained study staff twice, and a third time if the first two measures differed by more than 5 mmHg. An average of all three BP measures was used for analyses. We evaluated continuous measures of SBP and DBP, as well as elevated BP, defined as more than the 90th percentile accounting for age, sex, and height [44].

Covariates of interest included maternal factors in pregnancy: age, BMI, monthly income (in South African Rand), and socioeconomic (SES) status. SES was measured based on a composite score of asset ownership, household income, employment, and education adapted from items used in the South African Stress and Health Study and previously used in the DCHS [29,45]. Delivery and postpartum covariates included: birthweight (grams), length (cm), preterm birth (delivery <37 weeks’ gestation), low birthweight (<2500 g), high birthweight (>4000 g), mode of delivery (vaginal vs. cesarean-section), and duration of exclusive breastfeeding in months. Gestational age was estimated primarily using ultrasound in the second trimester. If ultrasound dating was not available, then symphysis-fundal height, recorded by trained clinical staff at study enrolment, or maternal recall of the last menstrual period was used for gestational age dating. Birth outcomes were captured by study staff who attended all deliveries. Information on pregnancy complications, including pre-eclampsia, eclampsia, hypertension, and gestational diabetes were collected by study staff during pregnancy and via chart review. However, the prevalence of pregnancy complications was low (<5%), thus they were not considered in this analysis [29]. Perceived household food insecurity was measured at the same study visit when cardiometabolic outcomes were assessed using the short form of the United States Department of Agriculture Household Food Security Scale, adapted for use in South Africa [46,47]. Physical activity was evaluated in all children starting at 7 years of age using the Physical Activity Questionnaire for Children, adapted for use in South Africa, and categorized as a number of times (0 through ≥4) a child engaged in physical activity in the last week [48]. Study data were collected and managed using REDCap [49,50].

### Statistical analysis

The goal of the statistical analysis was to evaluate if HIV-exposure status was associated with cardiometabolic outcomes in children at 5–8 years of age. We graphically described glucose metabolism, lipid levels, and BP by HIV-exposure status. To estimate associations between HIV-exposure status and cardiometabolic outcomes at 5–8 years of age, we used multivariable linear regression for continuous outcomes to estimate mean differences and multivariable log-binomial regression for binary outcomes to estimate risk ratios. Covariates that were associated with HIV exposure in pregnancy (e.g. maternal age) and that may predict cardiometabolic outcomes [e.g. child BMIZ score, age, sex, and height (for BP analyses)] were included as confounders. A number of factors occurring after pregnancy, such as duration of exclusive breastfeeding and child physical activity, could mediate the association between HIV exposure and cardiometabolic outcomes. The limited sample size precluded a formal mediation analysis [51]; however, we descriptively evaluated whether postnatal factors that differed by HIV-exposure status also predicted cardiometabolic outcomes at 5–8 years of age. Data were analyzed using Stata 15 (StataCorp Inc, College Station, Texas, USA).

## Results

We included 260 children (HEU *n* = 100, HIV-unexposed *n* = 160) with cardiometabolic outcome data and information from pregnancy. Overall, women tended to be overweight at study enrollment. HEU children had mothers that were older and more likely to be in the lowest SES quartile, compared with HIV-unexposed children (Table 1). There were no differences in gestational age at birth, weight, or length measures at birth, or birthweight. Women living with HIV were more likely to deliver via a cesarean section and to exclusively breastfeed for less time. There were no important differences between the full DCHS cohort and the sample included in this analysis with respect to sociodemographic, clinical, or HIV characteristics (Table S1 & Table S2). At the cardiometabolic visit, there were no differences in age [median age 7 (interquartile range 6,8) for both HEU and HIV-unexposed children] or physical activity by HIV-exposure status, but a higher proportion of HEU children were food insecure (Table S3).

**Table 1:** Characteristics of children HIV-unexposed and HIV-exposed, but uninfected in Drakenstein Child Health Study in pregnancy and delivery.

	HU	HEU	
	*n* = 160 (61.5)	*n* = 100 (38.5)	*P* value
During pregnancy			
Gestational age at enrollment, median (IQR)	23 (21, 26)	22 (20, 24)	0.03
Maternal age, median (IQR)	26 (22, 31)	30 (27, 34)	<0.01
Maternal BMI, median (IQR)	28 (25, 33)	29 (25, 34)	0.52
Socioeconomic status quartile^a^			0.30
Lowest	42 (26.3)	35 (35.0)	
Low-moderate	42 (26.3)	29 (29.0)	
Moderate-high	36 (22.5)	17 (17.0)	
High	40 (25.0)	19 (19.0)	
Household income per month			0.52
<R1000	74 (46.3)	39 (39.0)	
R1000–5000	70 (43.8)	50 (50.0)	
>R5000	16 (10.0)	11 (11.0)	
Delivery and postpartum			
Gestational age at delivery, median (IQR)	39 (38, 40)	39 (38, 40)	0.66
Birthweight, median (IQR)	3225 (2880, 3495)	3130 (2760, 3440)	0.17
Weight for age *z*-score, median (IQR)	−0.4 (−1.2, 0.2)	−0.4 (−1.2, 0.1)	0.83
Birth length (cm), median (IQR)	50 (48, 53)	50 (48, 52)	0.59
Duration of exclusive breastfeeding (months)^b^, median (IQR)	1.5 (0.9, 2.8)	0 (0, 3.1)	<0.01
Infant sex			0.20
Female	85 (53.1)	45 (45.0)	
Male	75 (46.9)	55 (55.0)	
Mode of delivery			0.03
Vaginal	125 (79.1)	67 (67.0)	
Cesarean-section	33 (20.9)	33 (33.0)	
Preterm birth (<37 weeks gestation)	16 (10.0)	15 (15.0)	0.23
Low birthweight (<2500 g)	13 (8.1)	9 (9.1)	0.79
High birthweight (>4000 g)	7 (4.4)	4 (4.0)	0.90

HEU, HIV-exposed but uninfected; HU, HIV-unexposed; IQR, interquartile range.

a Socioeconomic status based on a composite score of asset ownership, household income, employment, and education and divided into quartiles.

b Exclusive breastfeeding is defined as only feeding an infant breast or human milk. *P* values are based on the Mann–Whitney *U* test for continuous data and chi-squared tests for categorical data. Missing data: maternal age, *n* = 8; maternal BMI in pregnancy: *n* = 8, birthweight: *n* = 1, duration of exclusive breastfeeding: *n* = 1, low birthweight: *n* = 1, high birthweight: *n* = 1, mode of delivery: *n* = 2.

### Body composition and size

At 5–8 years of age, 18% of children had a BMIZ score more than 85th percentile, indicating overweight, and 19% of children had BMIZ score less than 15th percentile, indicating underweight. There were no differences in the proportion of children underweight or overweight by HIV-exposure status. In multivariable analyses of continuous *z*-scores, HEU children had lower average weight for age [WAZ; mean difference −0.35, 95% confidence interval (CI) −0.66, −0.05], height for age (HAZ; mean difference −0.29, 95% CI −0.56, −0.03) and somewhat lower BMI (BMIZ; mean difference −0.25, 95% CI −0.57, 0.06) *z*-scores, adjusted for maternal age in pregnancy and gestational age at enrollment (Table 2). HEU children also had somewhat lower fat mass (mean difference −0.46 kg, 95% CI −0.97, 0.04). There were no differences by HIV-exposures status among children in triceps skinfold thickness, percentage body fat, or percentage truncal fat.

**Table 2:** Body composition among HIV-unexposed and HIV-exposed, but uninfected children between 5 and 8 years of age.

	HU *n* = 160	HEU *n* = 100	Mean difference	
	Mean (SD)		(95% CI)	*P* value
Triceps skinfold thickness (mm)	9.7 (3.7)	9.2 (3.3)	−0.17 (−1.08, 0.73)^a^	0.71
Fat mass (kg)	5.3 (2.2)	4.8 (1.6)	−0.46 (−0.97, 0.04)^a^	0.07
Fat (%)	22.1 (4.9)	21.4 (3.7)	−0.54 (−1.65, 0.58)^a^	0.35
Truncal fat (%)	16.7 (4.9)	16.2 (3.6)	−0.37 (−1.52, 0.77)^a^	0.52
BMI *z*-score^b^	0.0 (1.2)	−0.2 (1.2)	−0.25 (−0.57, 0.06)^c^	0.11
WAZ-score^b^	0.0 (1.2)	−0.3 (1.1)	−0.35 (−0.66, −0.05)^c^	0.02
HAZ-score^b^	−0.1 (1.1)	−0.2 (0.9)	−0.29 (−0.56, −0.03)^c^	0.03

	*n* (%)		RR (95% CI)	
BMI *z*-score^b^ >85th percentile	33 (20.6)	15 (15.0)	0.67 (0.38, 1.20)^c^	0.18
BMI *z*-score^b^ <15th percentile	29 (18.1)	21 (21.0)	1.36 (0.79, 2.33)^c^	0.27

CI, confidence interval; HAZ, height for age; HEU, HIV-exposed but uninfected; HU, HIV-unexposed; RR, risk ratio; WAZ, weight for age.

a Adjusted for maternal age in pregnancy, gestational age at enrollment, child sex, and child age at cardiometabolic visit.

b *z*-scores based on child sex and age, according to WHO guidelines.

c Adjusted for maternal age in pregnancy and gestational age at enrollment. Missing data for fat mass, fat %, and truncal fat%: HU *n* = 3, HEU *n* = 3.

### Glucose metabolism

**Fig. 1:**
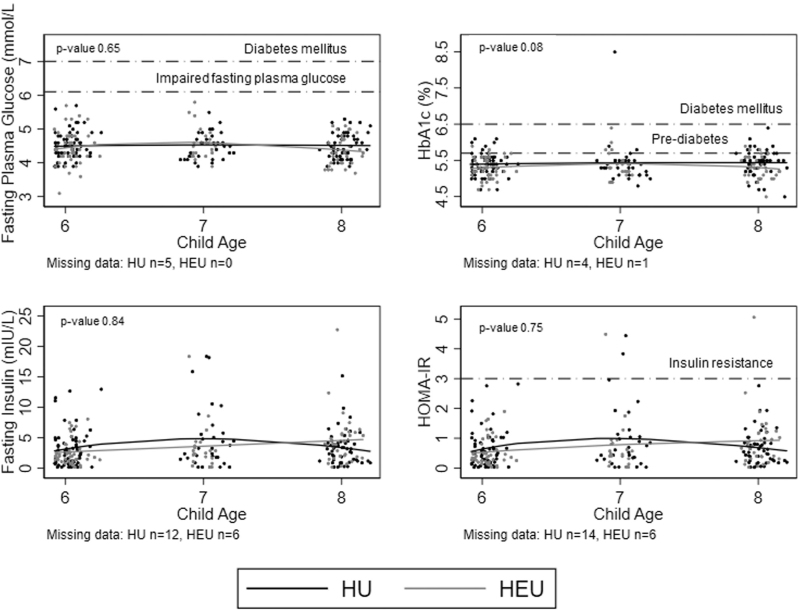
Glucose metabolism among HIV-unexposed children and HIV-exposed but uninfected children.

Overall, there were no cases of impaired fasting glucose or diabetes using fasting plasma glucose, and only one case of diabetes was identified using HbA1c (Table 3). Compared with fasting plasma glucose, HbA1c tended to overestimate prediabetes cases. Very few children had insulin resistance (*n* = 4). There was no evidence of differences in fasting plasma glucose, HbA1c, fasting insulin, or HOMA-IR values by HIV-exposure status (Table 3; Fig. 1).

**Table 3:** Cardiometabolic outcomes among HIV-unexposed and HIV-exposed, but uninfected children between 5 and 8 years of age.

	HU *n* = 160	HEU *n* = 100	Mean difference^a^	
	Mean (SD)		(95% CI)	*P* value
Total cholesterol (mmol/l)	3.8 (0.7)	3.8 (0.7)	0.07 (−0.12, 0.26)	0.49
HDL cholesterol (mmol/l)	1.3 (0.3)	1.3 (0.3)	−0.01 (−0.09, 0.08)	0.91
LDL cholesterol (mmol/l)	2.2 (0.6	2.2 (0.6	0.06 (−0.11, 0.23)	0.50
Triglycerides (mmol/l)	0.7 (0.3)	0.7 (0.2)	−0.02 (−0.09, 0.05)	0.55

	*n* (%)		RR (95% CI)	
Fasting plasma glucose				
Impaired fasting glucose (≥6.1–<7.0 mmol/l)	0 (0.0)	0 (0.0)	–	–
Diabetes (≥7.0 mmol/l)	0 (0.0)	0 (0.0)	–	–
HbA1c				
Prediabetes (5.7–6.4%)	16 (10.3)	9 (9.1)	1.00 (0.44, 2.28)	0.99
Diabetes (≥6.5%)	1 (0.6)	0 (0.0)	–	–
Insulin resistance (HOMA-IR ≥3.0)	2 (1.4)	2 (2.1)	1.97 (0.26, 14.98)	0.51
Blood pressure >90th percentile^b^	19 (12.0)	11 (11.2)	0.98 (0.47, 1.98)^c^	0.92

CI, confidence interval; HEU, HIV-exposed but uninfected; HU, HIV-unexposed; RR, risk ratio.

a Adjusted for child sex, child age, and BMIZ score at cardiometabolic visit, maternal age in pregnancy, and gestational age at enrollment.

b Defined as either SBP or DBP above the 90th percentile, based on age, height, and sex.

c Adjusted for child BMIZ score at cardiometabolic visit, maternal age in pregnancy, and gestational age at enrollment. Missing data: lipids: HU *n* = 3, HEU *n* = 2; fasting plasma glucose: HU *n* = 5; HbA1c: HU *n* = 4, HEU *n* = 1; fasting insulin: HU *n* = 12, HEU *n* = 6; HOMA IR: HU *n* = 14, HEU *n* = 6; blood pressure: HU *n* = 1, HEU *n* = 2.

Lines fit using fractional polynomial regression. *P* values compare mean differences by HIV exposure status and are adjusted for maternal age in pregnancy, gestational age at enrollment, child age, and BMIZ score at cardiometabolic visit.

### Lipids

The prevalence of high total and LDL cholesterol was less than 5%, 6% of children had high triglycerides, and 16% of children had low HDL cholesterol. There were no differences in mean lipid levels by HIV-exposure status (Table 3). When lipid levels were categorized as ‘normal’, ‘elevated’ and ‘high’ (or ‘normal’, ‘borderline’ and ‘low’ for HDL cholesterol), a higher proportion of HEU children had high TC (5 vs. 2.5%) and LDL cholesterol (6 vs. 2%) and low HDL cholesterol (20 vs. 14%), compared with HIV-unexposed children (Fig. S1). In multivariable analyses, these differences were not statistically different.

### Blood pressure

**Fig. 2:**
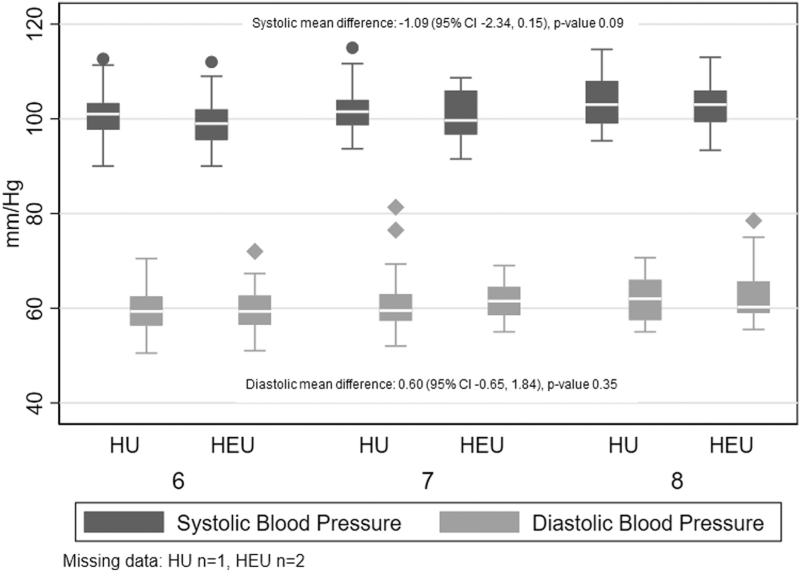
SBP and DBP at cardiometabolic visit by age and HIV exposure status.

Overall, 12% of children had BP more than 90th percentile, accounting for age, sex, and height. There were no differences in continuous measures of DBP or SBP, or BP more than the 90th percentile by HIV-exposure status (Table 3, Fig. 2).

Effect estimates compare mean differences blood pressure values by HIV exposure and are adjusted for maternal age, gestational age at enrollment, child sex, child age, sex, height, and BMIZ-score at cardiometabolic visit.

### Mediators

In exploratory analyses, we evaluated whether factors that occurred postnatally, such as size at birth, duration of breastfeeding, food insecurity status and physical activity differed by HIV-exposure status. For factors associated with HIV-exposure status, we evaluated if they also predicted cardiometabolic outcomes in multivariable analyses. HEU children had a shorter duration of exclusive breastfeeding (Table 1) and a higher proportion of food insecurity at the cardiometabolic visit (Table S3). However, the duration of breastfeeding and food insecurity status was not associated with body composition, glucose metabolism, lipids, or systolic blood (Table S4). Duration of exclusive breastfeeding was associated with a small increase in DBP. Overall, our findings suggest the duration of exclusive breastfeeding and food insecurity may be unlikely to strongly mediate any association between HIV exposure and cardiometabolic health in this population.

## Discussion

In a cohort of HEU and HIV-unexposed children in South Africa, we found overall low levels of impaired glucose metabolism, small body size, and dyslipidemia in children 5–8 years old. HEU children had somewhat lower height, weight, and fat mass compared with HIV-unexposed children. Although 12% of children had BP more than 90th percentile for their age, sex, and height, there was no evidence of differences between HEU and HIV-unexposed children. Despite HEU children being exclusively breastfed for shorter durations and having a higher proportion of food insecurity than HIV-unexposed children, in exploratory analyses we found little evidence that breastfeeding duration or food insecurity status was associated with clinically meaningful differences in cardiometabolic outcomes and, therefore, was likely to mediate the association between HIV-exposure and cardiometabolic outcomes. Taken together, these findings suggest that HEU children in this cohort have largely comparable cardiometabolic health with HIV-unexposed children at 5–8 years of age.

We observed small differences in body size and composition between HEU and HIV-unexposed children. As has been reported in other cohorts [11–14], HEU children had lower fat mass, and lower WAZ and HAZ scores, compared with HIV-unexposed children. Concerns about impaired growth among HEU children have long been noted [52]. However, more recent data suggest that within South Africa the differences in growth between HEU and HIV-unexposed children may be narrowing in the era of universal ART for women living with HIV, prolonged breastfeeding, and improved nutrition [53,54]. Our findings support the conclusion that observed differences in body size and composition within this cohort were small and not clinically meaningful at 5–8 years of age. For example, HEU children were on average less than half a standard deviation different in WAZ or HAZ *z*-scores than HIV-unexposed children. In addition, there were no differences by HIV-exposure status in children at the extremes of the BMI *z*-score distribution, at either more than 85th percentile or less than 15th percentile. BMI values among children in South Africa have been rising rapidly in the last 15 years in both HEU and HIV-unexposed children, with the largest increases observed in children 8–10 years of age [53,55,56]. Thus, while differences in growth or body composition by HIV-exposure status in this cohort were small and close to reference population mean values, on average, continued follow-up as children age will be essential.

A higher prevalence of dyslipidemia and impaired glucose metabolism has been noted among HEU children in sub-Saharan Africa and elsewhere [15,16,21–23]. In this cohort, we observed very few to no cases of prediabetes, diabetes, or insulin resistance and no differences by HIV-exposure status. Lipids levels were also fairly comparable between HEU and HIV-unexposed children; however, HEU children were somewhat more likely to have elevated levels of TC and LDL cholesterol and low HDL cholesterol. Although these differences were small and not statistically different, our sample size may have been underpowered to detect small differences. Given the young age of children (5–8 years) and the fact that HEU children were on average smaller and lighter than HIV-unexposed children, changes in lipid profiles and glucose metabolism for HEU and HIV-unexposed children should be monitored as children age into puberty and adolescence.

In this cohort, 12% of children overall had elevated BP by 5–8 years of age. High levels of elevated BP have been observed among other cohorts of South African children and represent an important public health concern [57–59]. Within this cohort, the risk of elevated BP and mean SBP or DBP did not differ by HIV-exposure status. HIV exposure has been linked to cardiac impairment in early childhood, most notably left ventricular diastolic dysfunction [60–66]. The potential clinical impact of HIV exposure on early cardiovascular function is still emerging. However, compared with HIV-unexposed children, an increased risk of hypertension has been noted among HEU children as young as 6 months of age [65]. In addition, reports from the PHACS cohort in the United States indicate that obese HEU adolescents are at higher risk of hypertension than obese HIV-unexposed adolescents [17]. These differences between our findings and published literature may reflect the young age, small sample size, or lack of obesity among children evaluated in this cohort.

### Conclusion

Overall, in a South African cohort of HEU and HIV-unexposed children we found low levels of glucose metabolism impairment, impaired growth, and adiposity, but higher levels of elevated BP at 5–8 years of age, with few differences by HIV-exposure status. Although our findings are reassuring for children exposed to HIV in-utero additional follow-up in larger longitudinal cohorts of HEU and HIV-unexposed children is needed to determine if small changes in body composition, growth, BP, and lipid levels persist or become more marked as children enter adolescence and puberty.

## Acknowledgements

We thank the mothers and their children for participating in the study and the study staff, the clinical and administrative staff of the Western Cape Government Health Department at Paarl Hospital and at the clinics for support of the study.

Author contributions: H.J.Z. obtained funding for the overall cohort; A.M.B. and J.P. obtained funding for the cardiometabolic pilot study. M.B., T.B., and H.J.Z. were responsible for data collection and data quality checks, in collaboration with R.M. A.M.B. drafted the article, with input from J.P., M.B., T.B., and H.J.Z. All authors helped to interpret the data analysis and critically reviewed the article.

Office of the Vice President of Research at Brown University; National Institutes of Mental Health (R00MH112413); The Bill & Melinda Gates Foundation, USA (grant numbers OPP1017641, OPP1017579); National Health and Medical Research Council (Australia) Investigator Grant to DB (1175744). H.J.Z. is supported by the South Africa-Medical Research Council. Research at MCRI is supported by the Victorian Government's Operational Infrastructure Support Program.

### Conflicts of interest

There are no conflicts of interest.

## Supplementary Material

## References

[1] UNAIDS. Prevaling against pandemics by putting people at the centre – World AIDS Day report 2020. Geneva, Switzerland: UNAIDS; 2020.

[2] Ramokolo V Goga AE Slogrove AL Powis KM. Unmasking the vulnerabilities of uninfected children exposed to HIV. *BMJ* 2019; 366:l4479. 31383646 10.1136/bmj.l4479 PMC6894435

[3] Slogrove AL Powis KM Johnson LF Stover J Mahy M. Estimates of the global population of children who are HIV-exposed and uninfected, 2000–18: a modelling study. *Lancet Global Health* 2019; 8:e67–e75. 31791800 10.1016/S2214-109X(19)30448-6 PMC6981259

[4] Evans C Jones CE Prendergast AJ. HIV-exposed, uninfected infants: new global challenges in the era of paediatric HIV elimination. *Lancet Infect Dis* 2016; 16:e92–e107. 27049574 10.1016/S1473-3099(16)00055-4

[5] Powis KM Slogrove AL Okorafor I Millen L Posada R Childs J. Maternal perinatal HIV infection is associated with increased infectious morbidity in HIV-exposed uninfected infants. *Pediatric Infect Dis J* 2019; 38:500–502. 10.1097/INF.0000000000002253 PMC6465126 30461574

[6] Arikawa S Rollins N Newell ML Becquet R. Mortality risk and associated factors in HIV-exposed, uninfected children. *Trop Med Int Health* 2016; 21:720–734. 27091659 10.1111/tmi.12695 PMC5021152

[7] Brennan AT Bonawitz R Gill CJ Thea DM Kleinman M Useem J. A meta-analysis assessing all-cause mortality in HIV-exposed uninfected compared with HIV-unexposed uninfected infants and children. *AIDS* 2016; 30:2351–2360. 27456985 10.1097/QAD.0000000000001211

[8] Newell ML Coovadia H Cortina-Borja M Rollins N Gaillard P Dabis F. Mortality of infected and uninfected infants born to HIV-infected mothers in Africa: a pooled analysis. *Lancet* 2004; 364:1236–1243. 15464184 10.1016/S0140-6736(04)17140-7

[9] Yeganeh N Watts DH Xu J Kerin T Joao EC Pilotto JH. Infectious morbidity, mortality and nutrition in HIV-exposed, uninfected, formula-fed infants: results from the HPTN 040/PACTG 1043 trial. *Pediatr Infect Dis J* 2018; 37:1271–1278. 29750766 10.1097/INF.0000000000002082 PMC6226320

[10] Slogrove AL Esser MM Cotton MF Speert DP Kollmann TR Singer J. A prospective cohort study of common childhood infections in South African HIV-exposed uninfected and HIV-unexposed Infants. *Pediatr Infect Dis J* 2017; 36:e38–e44. 28081048 10.1097/INF.0000000000001391 PMC5242219

[11] Lane CE Bobrow EA Ndatimana D Ndayisaba GF Adair LS. Determinants of growth in HIV-exposed and HIV-uninfected infants in the Kabeho Study. *Matern Child Nutr* 2019; 15:e12776. 30609287 10.1111/mcn.12776 PMC6667835

[12] Lane CE Widen EM Collins SM Young SL. HIV-exposed, uninfected infants in Uganda experience poorer growth and body composition trajectories than HIV-unexposed infants. *J Acquir Immune Defic Syndr* 2020; 85:138–147. 32604132 10.1097/QAI.0000000000002428 PMC7492413

[13] Ndiaye A Suneson K Njuguna I Ambler G Hanke T John-Stewart G. Growth patterns and their contributing factors among HIV-exposed uninfected infants. *Matern Child Nutr* 2021; 17:e13110. 33269548 10.1111/mcn.13110 PMC7988866

[14] Omoni AO Ntozini R Evans C Prendergast AJ Moulton LH Christian PS. Child growth according to maternal and child HIV status in Zimbabwe. *Pediatr Infect Dis J* 2017; 36:869–876. 28198792 10.1097/INF.0000000000001574 PMC5571879

[15] Claudio CC Patin RV Palchetti CZ Machado DM Succi RC Oliveira FL. Nutritional status and metabolic disorders in HIV-exposed uninfected prepubertal children. *Nutrition* 2013; 29:1020–1023. 23759262 10.1016/j.nut.2013.01.019

[16] Dirajlal-Fargo S Shan L Sattar A Bowman E Gabriel J Kulkarni M. Insulin resistance and intestinal integrity in children with and without HIV infection in Uganda. *HIV Med* 2020; 21:119–127. 31642582 10.1111/hiv.12808 PMC6980894

[17] Jao J Jacobson DL Yu W Borkowsky W Geffner ME McFarland EJ. A comparison of metabolic outcomes between obese HIV-exposed uninfected youth from the PHACS SMARTT Study and HIV-unexposed youth from the NHANES Study in the US. *J Acquir Immune Defic Syndr* 2019; 81:319–327. 30844997 10.1097/QAI.0000000000002018 PMC6565481

[18] Fourman LT Pan CS Zheng I Gerard ME Sheehab A Lee H. Association of in utero HIV exposure with obesity and reactive airway disease in HIV-negative adolescents and young adults. *J Acquir Immune Defic Syndr* 2020; 83:126–134. 31738195 10.1097/QAI.0000000000002235 PMC7237070

[19] Kirmse B Hobbs CV Peter I Laplante B Caggana M Kloke K. Abnormal newborn screens and acylcarnitines in HIV-exposed and ARV-exposed infants. *Pediatr Infect Dis J* 2013; 32:146–150. 22935866 10.1097/INF.0b013e31827030a6 PMC4648253

[20] Kirmse B Yao TJ Hofherr S Kacanek D Williams PL Hobbs CV. Acylcarnitine profiles in HIV-exposed, uninfected neonates in the United States. *AIDS Res Hum Retroviruses* 2016; 32:339–348. 26548585 10.1089/aid.2015.0112 PMC4817565

[21] Jao J Powis KM Kirmse B Yu C Epie F Nshom E. Lower mitochondrial DNA and altered mitochondrial fuel metabolism in HIV-exposed uninfected infants in Cameroon. *AIDS* 2017; 31:2475–2481. 28926411 10.1097/QAD.0000000000001647 PMC5680102

[22] Jao J Balmert LC Sun S Qiu Y Kraus TA Kirmse B. Distinct cord blood C-peptide, adipokine, and lipidomic signatures by in utero HIV exposure. *Pediatr Res* 2021; 92:233–241. 34446848 10.1038/s41390-021-01705-1 PMC8881568

[23] Jao J Kirmse B Yu C Qiu Y Powis K Nshom E. Lower preprandial insulin and altered fuel use in HIV/antiretroviral-exposed infants in Cameroon. *J Clin Endocrinol Metab* 2015; 100:3260–3269. 26133363 10.1210/JC.2015-2198 PMC4570172

[24] Eckard AR Kirk SE Hagood NL. Contemporary issues in pregnancy (and Offspring) in the current HIV era. *Curr HIV AIDS Rep* 2019; 16:492–500. 31630334 10.1007/s11904-019-00465-2 PMC6938215

[25] le Roux SM Abrams EJ Nguyen K Myer L. Clinical outcomes of HIV-exposed, HIV-uninfected children in sub-Saharan Africa. *Trop Med Int Health* 2016; 21:829–845. 27125333 10.1111/tmi.12716

[26] Zar HJ Barnett W Myer L Stein DJ Nicol MP. Investigating the early-life determinants of illness in Africa: the Drakenstein Child Health Study. *Thorax* 2015; 70:592–594. 25228292 10.1136/thoraxjnl-2014-206242 PMC5107608

[27] Republic of South Africa. Statistics of South Africa, mid-year population estimates. *Statistical release*. Pretoria, South Africa: Republic of South Africa Department: South Africa Statistics; 2019.

[28] Republic of South Africa Department of Health. Guidelines for maternity care in South Africa. Pretoria, South Africa: Department of Health; 2015.

[29] Zar HJ Pellowski JA Cohen S Barnett W Vanker A Koen N. Maternal health and birth outcomes in a South African birth cohort study. *PLoS One* 2019; 14:e0222399–e1222399. 31751344 10.1371/journal.pone.0222399 PMC6874071

[30] Meintjes G Black J Conradie F Dlamini S Maartens G Manzini TC. Southern African HIV Clinicians Society adult antiretroviral therapy guidelines: update on when to initiate antiretroviral therapy. *Southern Afr J HIV Med* 2015; 16:428. 10.4102/sajhivmed.v16i1.428 PMC5843256 29568598

[31] Meintjes G Conradie JBF Cox V Dlamini S Fabian J Maartens G. Adult antiretroviral therapy guidelines 2014. *Southern Afr J HIV Med* 2014; 15:121–143.

[32] WHO. Consolidated guidelines on the use of antiretroviral drugs for treating and preventing HIV infection: recommendations for a public health approach. Geneva, Switzerland: World Health Organization; 2013. 24716260

[33] Pellowski J Wedderburn C Stadler JAM Barnett W Stein D Myer L. Implementation of prevention of mother-to-child transmission (PMTCT) in South Africa: outcomes from a population-based birth cohort study in Paarl, Western Cape. *BMJ Open* 2019; 9:e033259. 10.1136/bmjopen-2019-033259 PMC6924830 31843848

[34] Talma H Chinapaw MJ Bakker B HiraSing RA Terwee CB Altenburg TM. Bioelectrical impedance analysis to estimate body composition in children and adolescents: a systematic review and evidence appraisal of validity, responsiveness, reliability and measurement error. *Obes Rev* 2013; 14:895–905. 23848977 10.1111/obr.12061

[35] Wells JC. Toward body composition reference data for infants, children, and adolescents. *Adv Nutr* 2014; 5:320S–329S. 24829484 10.3945/an.113.005371 PMC4013189

[36] Browning LM Mugridge O Chatfield MD Dixon AK Aitken SW Joubert I. Validity of a new abdominal bioelectrical impedance device to measure abdominal and visceral fat: comparison with MRI. *Obesity* 2010; 18:2385–2391. 20360757 10.1038/oby.2010.71 PMC3308203

[37] World Health Organization. Training course on child growth assessment. Geneva, Switzerland: WHO; 2008.

[38] World Health Organization, United Nations Children's Fund. WHO child growth standards and the identification of severe acute malnutrition in infants and children. A Joint Statement. Geneva, Switzerland: World Health Organization; 2009. 24809116

[39] World Health Organization. Use of glycated hemoglobin (HbA1c) in the diagnosis of diabetes mellitus. *Abbreviated report of a WHO consultation 2011*. Geneva, Switzerland: World Health Organization; 2011. 26158184

[40] World Health Organization. Definition and diagnosis of diabetes mellitus and intermediate hyperglycemia: report of a WHO/IDF consultation. Geneva, Switzerland: World Health Organization; 2006.

[41] Matthews DR Hosker JP Rudenski AS Naylor BA Treacher DF Turner RC. Homeostasis model assessment: insulin resistance and beta-cell function from fasting plasma glucose and insulin concentrations in man. *Diabetologia* 1985; 28:412–419. 3899825 10.1007/BF00280883

[42] Tresaco B Bueno G Pineda I Moreno LA Garagorri JM Bueno M. Homeostatic model assessment (HOMA) index cut-off values to identify the metabolic syndrome in children. *J Physiology Biochem* 2005; 61:381–388. 10.1007/BF03167055 16180336

[43] Services UDoHaH. Expert panel on integrated guidelines for cardiovascular health and risk reduction in children and adolescents. Bethesda, Maryland: NIH: National Heart, Lung, Blood Institute; 2012.

[44] Flynn JT Kaelber DC Baker-Smith CM Blowey D Carroll AE Daniels SR. Clinical practice guideline for screening and management of high blood pressure in children and adolescents. *Pediatrics* 2017; 140:e20171904. 28827377 10.1542/peds.2017-1904

[45] Myer L Stein DJ Grimsrud A Seedat S Williams DR. Social determinants of psychological distress in a nationally-representative sample of South African adults. *Soc Sci Med* 2008; 66:1828–1840. 18299167 10.1016/j.socscimed.2008.01.025 PMC3203636

[46] Pellowski JA Barnett W Kuo CC Koen N Zar HJ Stein DJ. Investigating tangible and mental resources as predictors of perceived household food insecurity during pregnancy among women in a South African birth cohort study. *Soc Sci Med* 2017; 187:76–84. 28666232 10.1016/j.socscimed.2017.06.022 PMC5580987

[47] Bickel D Nord M Price C Hamilton W Cook J. United States Department of Agriculture (USDA) guide to measuring household food security. Alexandria, VA: USDA; 2000.

[48] Crocker PR Bailey DA Faulkner RA Kowalski KC McGrath R. Measuring general levels of physical activity: preliminary evidence for the Physical Activity Questionnaire for Older Children. *Med Sci Sports Exerc* 1997; 29:1344–1349. 9346166 10.1097/00005768-199710000-00011

[49] Harris PA Taylor R Thielke R Payne J Gonzalez N Conde JG. Research electronic data capture (REDCap)—a metadata-driven methodology and workflow process for providing translational research informatics support. *J Biomed Inform* 2009; 42:377–381. 18929686 10.1016/j.jbi.2008.08.010 PMC2700030

[50] Harris PA Taylor R Minor BL Elliott V Fernandez M O’Neal L. The REDCap consortium: building an international community of software platform partners. *J Biomed Inform* 2019; 95:103208. 31078660 10.1016/j.jbi.2019.103208 PMC7254481

[51] VanderWeele TJ. A three-way decomposition of a total effect into direct, indirect, and interactive effects. *Epidemiology* 2013; 24:224–232. 23354283 10.1097/EDE.0b013e318281a64e PMC3563853

[52] Jao J Abrams EJ. Metabolic complications of in utero maternal HIV and antiretroviral exposure in HIV-exposed infants. *Pediatr Infect Dis J* 2014; 33:734–740. 24378947 10.1097/INF.0000000000000224 PMC4055505

[53] le Roux SM Abrams EJ Donald KA Brittain K Phillips TK Nguyen KK. Growth trajectories of breastfed HIV-exposed uninfected and HIV-unexposed children under conditions of universal maternal antiretroviral therapy: a prospective study. *Lancet Child Adolesc Health* 2019; 3:234–244. 30773459 10.1016/S2352-4642(19)30007-0

[54] Bengtson AM le Roux SM Phillips TK Brittain K Zerbe A Madlala HP. Relationship between prepregnancy maternal body mass index and infant weight trajectories in HIV-exposed and HIV-unexposed infants. *Paediatr Perinat Epidemiol* 2022; 36:536–547. 34859468 10.1111/ppe.12825 PMC9163208

[55] Di Cesare M Sorić M Bovet P Miranda JJ Bhutta Z Stevens GA. The epidemiological burden of obesity in childhood: a worldwide epidemic requiring urgent action. *BMC Med* 2019; 17:212. 31760948 10.1186/s12916-019-1449-8 PMC6876113

[56] Sartorius B Sartorius K Taylor M Aagaard-Hansen J Dukhi N Day C. Rapidly increasing body mass index among children, adolescents and young adults in a transitioning population, South Africa, 2008–15. *Int J Epidemiol* 2018; 47:2099. 30376046 10.1093/ije/dyy248 PMC6280934

[57] Matjuda EN Engwa GA Anye SNC Nkeh-Chungag BN Goswami N. Cardiovascular risk factors and their relationship with vascular dysfunction in South African Children of African Ancestry. *J Clin Med* 2021; 10:354. 33477761 10.3390/jcm10020354 PMC7832309

[58] Matjuda EN Engwa GA Letswalo PB Mungamba MM Sewani-Rusike CR Nkeh-Chungag BN. Association of hypertension and obesity with risk factors of cardiovascular diseases in children aged 6-9 years old in the Eastern Cape Province of South Africa. *Children (Basel)* 2020; 7:25. 32231008 10.3390/children7040025 PMC7230217

[59] Houle B Rochat TJ Newell ML Stein A Bland RM. Breastfeeding, HIV exposure, childhood obesity, and prehypertension: a South African cohort study. *PLoS Med* 2019; 16:e1002889. 31454346 10.1371/journal.pmed.1002889 PMC6711496

[60] Lipshultz SE Sasaki N Thompson B Eidem BW Cheng I Colan SD. Left ventricular diastolic dysfunction in HIV-uninfected infants exposed in utero to antiretroviral therapy. *AIDS* 2020; 34:529–537. 31764073 10.1097/QAD.0000000000002443 PMC8806162

[61] Lipshultz SE Miller TL Wilkinson JD Scott GB Somarriba G Cochran TR. Cardiac effects in perinatally HIV-infected and HIV-exposed but uninfected children and adolescents: a view from the United States of America. *J Int AIDS Soc* 2013; 16:18597. 23782480 10.7448/IAS.16.1.18597 PMC3687072

[62] Lipshultz SE Shearer WT Thompson B Rich KC Cheng I Orav EJ. Cardiac effects of antiretroviral therapy in HIV-negative infants born to HIV-positive mothers: NHLBI CHAART-1 (National Heart, Lung, and Blood Institute Cardiovascular Status of HAART Therapy in HIV-Exposed Infants and Children cohort study). *J Am Coll Cardiol* 2011; 57:76–85. 21185505 10.1016/j.jacc.2010.08.620 PMC3243620

[63] Cade WT Waggoner AD Hubert S Krauss MJ Singh GK Overton ET. Reduced diastolic function and left ventricular mass in HIV-negative preadolescent children exposed to antiretroviral therapy in utero. *Aids* 2012; 26:2053–2058. 22874520 10.1097/QAD.0b013e328358d4d7 PMC3641749

[64] Guerra V Leister EC Williams PL Starc TJ Lipshultz SE Wilkinson JD. Long-term effects of in utero antiretroviral exposure: systolic and diastolic function in HIV-exposed uninfected youth. *AIDS Res Hum Retroviruses* 2016; 32:621–627. 26794032 10.1089/aid.2015.0281 PMC4931731

[65] GarcÍa-Otero L LÓpez M GoncÉ. A Fortuny C Salazar L Valenzuela-Alcaraz B. Cardiac remodeling and hypertension in HIV-uninfected infants exposed in utero to antiretroviral therapy. *Clin Infect Dis* 2021; 73:586–593. 33471090 10.1093/cid/ciab030

[66] Martins P Pires A Albuquerque ME Oliveira-Santos M Santos J Sena C. Myocardial peak systolic velocity-a tool for cardiac screening of HIV-exposed uninfected children. *Eur J Pediatr* 2020; 179:395–404. 31761972 10.1007/s00431-019-03477-7

