# Supplementary material for: In-utero HIV exposure and cardiometabolic health among children 5–8 years: findings from a prospective birth cohort in South Africa
Source: AIDS. 2022 Oct 19;37(1):173–82. doi: 10.1097/QAD.0000000000003412 (PMC9751971; doi:10.1097/QAD.0000000000003412)
Supplement: Supplementary file 1 [file aids-37-173-s001.docx]

**Figure S1. Lipid categories among HIV-unexposed (HU) and HIV-exposed, but uninfected (HEU) children at 5-8 years of age.** Effect estimates compare the risk of an abnormal (vs normal) lipid level by HIV exposure status and are adjusted for maternal age in pregnancy, gestational age at enrollment, child age and BMIZ score at cardiometabolic visit. Missing data: HU n=3, HEU n=2. LDL: low-density lipoprotein; HDL: high-density lipoprotein. All units in mmol/L.


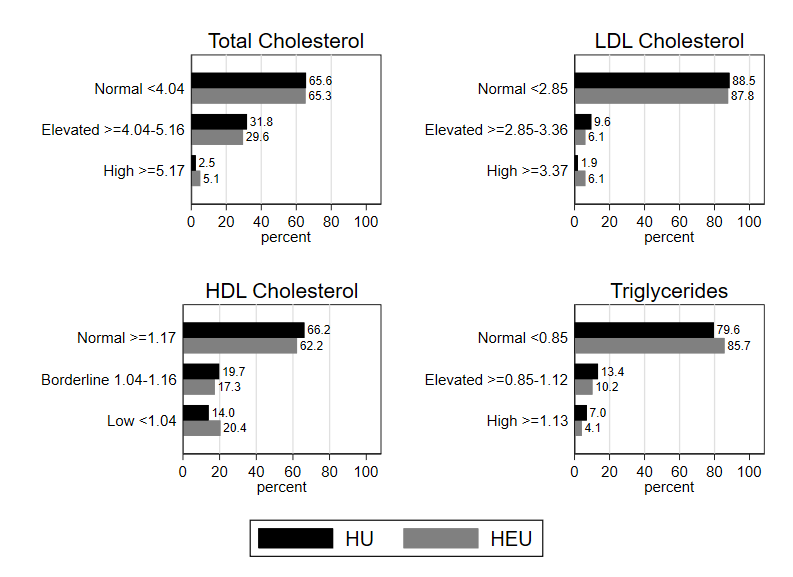


RR 1.04 (95% CI 0.73, 1.48)

p-value 0.85

RR 1.15 (95% CI 0.56, 2.36)

p-value 0.70

RR 0.70 (95% CI 0.39, 1.27)

p-value 0.24

RR 0.98 (95% CI 0.70, 1.39)

p-value 0.93
