# Supplementary material for: In-utero HIV exposure and cardiometabolic health among children 5–8 years: findings from a prospective birth cohort in South Africa
Source: AIDS. 2022 Oct 19;37(1):173–82. doi: 10.1097/QAD.0000000000003412 (PMC9751971; doi:10.1097/QAD.0000000000003412)
Supplement: Supplementary file 2 [file aids-37-173-s002.docx]

**Supplemental Tables and Figures**

**Table S1. Comparison of sociodemographic characteristics between participants in the cardiometabolic sub-study and the full Drakenstein Child Health Study Cohort**

|  | **Cardiometabolic**  **Sub-Study** | **Full Cohort** |
| --- | --- | --- |
|  | **N=260** | **n=1137** |
| **During pregnancy** |  |  |
| Maternal age at enrollment, median (IQR) | 27.7 (23.1, 32.3) | 25.8 (22, 30.8) |
| Maternal body mass index (BMI) , median (IQR) | 28.3 (24.6, 33.7) | 26 (22, 32) |
| Socioeconomic status quartile |  |  |
| Lowest | 77 (29.6) | 274 (24.1) |
| Low-moderate | 71 (27.3) | 293 (25.8) |
| Moderate-high | 53 (20.4) | 288 (25.3) |
| High | 59 (22.7) | 282 (24.8) |
| Household income per month |  |  |
| < R1000 | 113 (43.5) | 430 (37.8) |
| R1000-5000 | 120 (46.2) | 553 (48.6) |
| >R5000 | 27 (10.4) | 154 (13.6) |
| **Delivery & postpartum** |  |  |
| Gestational age at delivery, median (IQR) | 39 (38, 40) | 39 (37, 40) |
| Birthweight, median (IQR) | 3.1 (2.8, 3.5) | 3.1 (2.7, 3.4) |
| Weight for age z-score, median (IQR) | -0.4 (-1.2, 0.1) | -0.5 (-1.3, 0.1) |
| Duration of exclusive breastfeeding (months), median (IQR) | 1 (0, 2.8) | 1.6 (0.9, 3.0) |
| Infant sex |  |  |
| Female | 130 (50.0) | 554 (41.4) |
| Male | 130 (50.0) | 589 (51.6) |
| Preterm birth (<37 weeks gestation) | 32 (12.3) | 191 (16.7) |
| Low birthweight (<2500 grams) | 22 (8.5) | 174 (15.2) |

**Table S2. Comparison of HIV characteristics during pregnancy between participants in the cardiometabolic sub-study and the full Drakenstein Child Health Study Cohort**

|  | **Cardiometabolic Sub-Study** | **Full Cohort** |
| --- | --- | --- |
|  | **N=100** | **N=244** |
| On ART in pregnancy | **N(%)** | **N(%)** |
| No | 11 (11) | 2 (1) |
| Yes | 83 (83) | 236 (97) |
| Not available | 5 (5) | 5 (2) |
| Viral Load |  |  |
| Detectable (.= 40 copies/mL) | 13 (13) | 47 (19) |
| Undetectable (<40 copies/mL) | 51 (51) | 101 (46) |
| Not available | 36 (36) | 95 (39) |
| CD4 cell count, median (IQR) | 416 (287, 618) | 411 (286–609) |

| **Table S3.** Characteristics of children at cardiometabolic assessment study visit or later, by HIV exposure status | | | |
| --- | --- | --- | --- |
|  | **HU** | **HEU** |  |
|  | **n=160 (61.5)** | **n=100 (38.5)** | **p-value** |
| Child age, median (IQR) | 7 (6, 8) | 7 (6, 8) | 0.52 |
| Food insecurity^1^ |  |  | 0.09 |
| No | 139 (86.9) | 79 (79.0) |  |
| Yes | 21 (13.1) | 21 (21.0) |  |
| Physical activity in the last week (among children reaching 7 years of age)^2^ | n=128 | n=78 | 0.50 |
| None | 41 (32.3) | 20 (26.3) |  |
| 1 time | 17 (13.4) | 14 (18.4) |  |
| 2-3 times | 50 (39.4) | 34 (44.7) |  |
| ≥4 times | 19 (15.0) | 8 (10.5) |  |
| ^1^ Food insecurity measures using an adapted version of the short form of the United States Department of Agriculture Household Food Security Scale; ^2^ physical activity measured using an adapted version of the Physical Activity Questionnaire for Children (PAQ-C). P-values based on the Mann Whitney U test for continuous data and chi-squared tests for categorical data. Missing data: none. | | | |

| **Table S4.** Associations of potential mediators with cardiometabolic outcomes | | | |  |
| --- | --- | --- | --- | --- |
| **Potential Mediator** | **Food Insecurity** | | **Months exclusively breastfeeding** | |
|  | **Mean difference** | **p-value** | **Mean difference** | **p-value** |
| **Cardiometabolic outcome** | **(95% CI)** |  | **(95% CI)** |  |
| Body composition & size |  |  |  |  |
| Fat mass, (kg)^1^ | -0.11 (-0.44, 0.22) | 0.50 | 0.01 (-0.05, 0.08) | 0.66 |
| BMI z-score^2^ | -0.09 (-0.49, 0.32) | 0.68 | -0.02 (-0.10, 0.06) | 0.61 |
| WAZ-score^2^ | -0.14 (-0.53, 0.24) | 0.46 | -0.04 (-0.11, 0.03) | 0.3 |
| HAZ-score^2^ | -0.15 (-0.49, 0.18) | 0.37 | -0.04 (-0.10, 0.02) | 0.23 |
| Blood pressure |  |  |  |  |
| Systolic, mm/Hg^3^ | -0.46 (-2.05, 1.13) | 0.57 | 0.23 (-0.07, 0.52) | 0.13 |
| Diastolic, mm/Hg^3^ | -0.45 (-1.14, 2.03) | 0.58 | 0.35 (0.06, 0.64) | 0.02 |
| Lipids |  |  |  |  |
| Total cholesterol, mmol/L^1^ | 0.03 (-0.21, 0.27) | 0.81 | 0.02 (-0.02, 0.07) | 0.36 |
| HDL cholesterol, mmol/L^1^ | -0.05 (-0.16, 0.05) | 0.33 | 0.01 (-0.01, 0.02) | 0.62 |
| LDL cholesterol, mmol/L^1^ | 0.12 (-0.09, 0.34) | 0.27 | 0.02 (-0.03, 0.06) | 0.45 |
| Triglycerides, mmol/L^1^ | -0.05 (-0.14, 0.03) | 0.23 | -0.01 (-0.02, 0.01) | 0.67 |
| Glucose Metabolism |  |  |  |  |
| Fasting plasma glucose, mmol/L^1^ | 0.06 (-0.08, 0.20) | 0.39 | -0.01 (-0.03, 0.02) | 0.56 |
| HbA1c, %^1^ | -0.02 (-0.15, 0.11) | 0.75 | -0.01 (-0.04, 0.01) | 0.25 |
| Fasting Insulin, mIU/L^1^ | -0.04 (-1.21, 1.12) | 0.94 | -0.02 (-0.24, 0.20) | 0.86 |
| HOMA-IR^1^ | 0.01 (-0.25, 0.26) | 0.98 | -0.01 (-0.05, 0.04) | 0.75 |
| ^1^ Estimates adjusted for child sex, child age, HIV exposure status, BMIZ score at cardiometabolic visit, maternal age in pregnancy, and gestational age at enrollment. ^2^ z-scores based on child sex and age, according to WHO guidelines; estimates adjusted for HIV exposure status, maternal age in pregnancy and gestational age at enrollment. ^3^Estimates adjusted for child sex, child age, child height, HIV exposure status, BMIZ score at cardiometabolic visit, maternal age in pregnancy, and gestational age at enrollment | | | | |
